# Notes and News

**Published:** 1902-03-08

**Authors:** 


### Notes and News.

Lord Sandhurst has accepted the post of chairman of the weekly board of governors of Middlesex Hospital.

We have no doubt that when its affairs are placed in the hands of a responsible committee of management, Addenbrooke's Hospital will soon issue a report to the governors each year recording in an interesting way the work of the managers, the progress of the institution, its requirements and needs, in view of the claims of the community and the efficient treatment and comfort of the inmates. At present this hospital does not issue such a report. The absence of such an annual statement, attractively and forcibly written, reveals somnolence in the institution immediately concerned, and creates indifference and sometimes even despair in the minds of those governors who turn to the pages of a so-called report which is prepared upon the ancient lines now in vogue at Cambridge.

The authorities of the Brompton Hospital for Consumption are losing no time in the provision of the sanatorium for the treatment of consumption at Camberley, in accordance with the decision arrived at by the governors a short time ago. About 20 acres of land have been purchased near Camberley, in the heart of the pine country. The building, which has been designed by Mr. E. T. Hall, F.R.I.B.A., will provide accommodation for 100 patients, who will be of the non-paying class. The sanatorinm will be two storeys in height, and will consist of four wings or pavillions, radiating to a central block. The majority of the patients' bedrooms will be single. There will be a few double rooms, and a few for three beds, to provide for cases of an exceptional character. All the patients' rooms will be made to face south, south-south-west, or south-south-east. Special attention has been given to the question of ventilation, with the object of admitting the greatest possible amount of fresh air without inconvenience or risk to patients. The windows will be so constructed that patients can be wheeled out in their beds if necessary, and special blinds have been designed to afford shelter from sun and rain.

Attention is again being drawn to the insanitary condition of the House of Commons.

Pensions are to be established and beds endowed at the British Home for Incurables in commemoration of Queen Alexandra's Coronation. This charity was the first to receive her patronage in this country.

In order to facilitate the diagnosis of small-pox, the London County Council have issued a list of medical men in different parts of London who are willing to be consulted in doubtful cases of chicken-pox, the Council paying the necessary fee. The list can be procured from the clerk of the Council, County Hall, Spring Gardens, S.W.

Mr. Bernard Shaw has propounded the most amusing and original suggestion to explain the reason why seven nurses at the Mile End Infirmary, who were unrevaccinated, caught small-pox, whilst all the revaccinated nurses escaped. He suggests that "the presence in the infirmary of a number of nurses suffering from variolous eruptions on the arm produced, not only the outbreak among the unrevaccinated nurses, but also among the patients. Of course it is needless to go into the medical aspect of his suggestion here, but we would point out that at any rate Mr. Shaw has raised an argument in favour of simultaneous and universal vaccination, for if his argument is correct, we must protect ourselves against the vaccinated who are amongst us everywhere.

Owing to the action of the Medical Officer of Health for Halifax, seven firms in that town were proceeded against for selling preserved peas containing sulphate of copper. After a consideration of the fact that the amount of sulphate of copper used was not large enough to be a real danger to health, that no definite regulations as to the addition of preservatives to food had yet been made, that the prohibition or otherwise depended upon the degree of vigilance of individual medical officers, and so was unequal in its effects in different localities, and that the public demanded vegetables of good colour, the magistrates fined each firm five shillings. The chairman expressed his opinion that he did not regard the sulphate of copper as used a danger to health, but he said that he must administer the law as he found it.

We learn that the arrangements between St. Bartholomew's Hospital and Christ's Hospital are not generally understood. Mr. Haig Brown, an Almoner of Christ's Hospital, explains the situation to be that the Almoners of St. Bartholomew's Hospital offered to purchase about one and a half acres of the vacated site of Christ's Hospital for £117,000. The Almoners of Christ's Hospital considering the value to be more than twice that sum, refused the offer. In the end, Lord Balfour of Burleigh was appointed arbitrator, and he assessed the value at £238,781. It is not unnatural that the Almoners of Christ's Hospital endeavoured to secure good value for their trust, nor that the Almoners of St. Bartholomew's Hospital should strive to obtain the best bargain for their charity. Whichever gains by the transaction the public benefits.

